# Hydroethanolic Extract of Defatted *Buchholzia coriacea* Seeds Alleviates Tamoxifen-Induced Hepatic Triglyceride Accumulation, Inflammation and Oxidative Distress in Rat

**DOI:** 10.3390/medicines9010001

**Published:** 2021-12-24

**Authors:** Ayokanmi Ore, Abideen Idowu Adeogun, Oluseyi Adeboye Akinloye

**Affiliations:** 1 Department of Biochemistry, College of Biosciences, Federal University of Agriculture, Abeokuta 110101, Nigeria; 2 Biochemistry Division, Department of Chemical Sciences, Faculty of Natural Sciences, Ajayi Crowther University, Oyo 200284, Nigeria; 3 Department of Chemistry, College of Physical Sciences, Federal University of Agriculture, Abeokuta 110101, Nigeria; abuaisha2k3@yahoo.com

**Keywords:** tamoxifen, drug induced liver injury, hepatic steatosis, hepatocyte ballooning, inflammation, oxidative stress, antioxidants, *Buchholzia coriacea* seed, immunohistochemistry, rat

## Abstract

**Background:** Tamoxifen (TMX) has proven to be effective in the prevention and treatment of breast cancer. However, long-term use of TMX is associated with hepatic steatosis, oxidative liver injury and hepatocarcinoma. *Buchholzia coriacea* seeds (BCS) have been widely applied in traditional medicine due to their nutritional and therapeutic potentials. This study investigates the protective effect of hydroethanolic extract of (defatted) *B. coriacea* seeds (HEBCS) against TMX–induced hepatotoxicity in rats. **Methods:** Thirty-six (36) male albino rats were divided into six groups (*n* = 6/group). Group I served as control. Group II received 50 mg/kg/day TMX orally (p.o.) (TMX) for 21 days, group III received TMX plus 125 mg/kg/d HEBCS p.o. (HEBCS 125) for 21 days, group IV received TMX plus 250 mg/kg/d HEBCS p.o. (HEBCS 250) for 21 days and rats in group V and VI received HEBCS 125 and HEBCS 250 respectively for 21 days. **Results:** Compared with the control, TMX caused a significant increase (*p* < 0.05) in serum hepatic function biomarkers: alanine aminotransferase, aspartate aminotransferase and alkaline phosphatase by 57%, 60% and 68% respectively. TMX also caused a significant increase in hepatic triglycerides level by 166% when compared with control and a significant decrease in serum HDL-cholesterol level by 37%. Compared with control, hepatic marker of inflammation, tumour necrosis factor alpha (TNF-α) increased significantly by 220%, coupled with significant increase in expression of interleukin 6 and cyclooxygenase 2. There was also significant increase in levels of Biomarkers of oxidative stress, nitric oxide, malondialdehyde and protein carbonyls in the TMX group by 89%, 175% and 114% respectively when compared with the control. Hepatic antioxidants, reduced glutathione (GSH) level and activities of superoxide dismutase (SOD), catalase (CAT), glutathione S-transferase (GST) and glutathione peroxidase (GSH-Px) decreased significantly in the TMX group by 35%, 67%, 41%, 59% and 53% respectively when compared with the control. However, HEBCS at 250 mg/kg significantly protected against TMX–induced hepatotoxicity by decreasing hepatic triglyceride content, serum hepatic function biomarkers, hepatic inflammation and oxidative stress with significant improvement in hepatic antioxidant system. Histopathological findings show that HEBCS alleviate TMX–induced hepatocyte ballooning. **Conclusions:** Current data suggest that HEBCS protected against TMX–induced hepatotoxicity in rats. HEBCS may be useful in managing TMX–induced toxicities in breast cancer patients. It may also be helpful against other forms of liver injury involving steatosis, inflammation, free radicals, and oxidative damage.

## 1. Introduction

Hepatic diseases represent a major public health concern worldwide. Over the past two decades, cases of liver disease have increased to become one of the leading causes of death [1]. According to the global burden of disease, liver disease is estimated to account for up to two million deaths per year [2]. Drug-induced liver injury (DILI) refers to unexpected harmful effects of drugs on the liver, which includes damage to hepatocytes and other hepatic cells [3]. DILI can range from mild elevation of blood activities of aminotransferases to acute liver failure (ALF), leading to liver transplantation or death [4]. Histological pattern or phenotypes of DILI include cholestasis, acute hepatitis, chronic hepatitis, cholestatic hepatitis, granulomatous hepatitis, steatosis and steatohepatitis [5,6].

**Figure 1:**
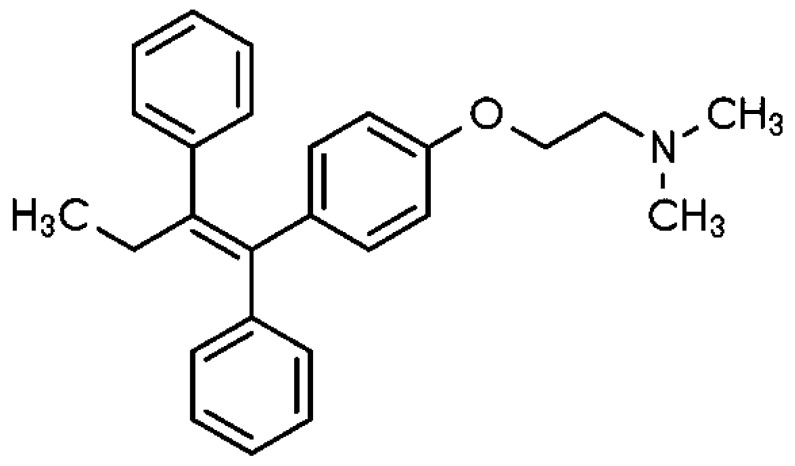
Chemical structure of Tamoxifen.

Tamoxifen (TMX), 1-[4-(2-dimethyl-aminoethoxy)phenyl]-1,2-diphenyl-1-butene; Figure 1, is a first-line chemotherapy in the prevention and treatment of estrogen-receptor-positive breast cancer [7]. Tamoxifen is a pro-drug and therefore undergoes metabolic bioactivation. TMX is metabolized to 4-hydroxytamoxifen by CYP2D6, and subsequently converted into endoxifen by CYP3A4/5. Endoxifen is the most active metabolite, which is regarded as even more potent compared to tamoxifen itself [8,9].

Generally, TMX usage has led to an increase in survival rate in breast cancer patients. However, metabolic activation of TMX by cytochrome P450 mixed-function oxidases in liver results in the production of free radicals and reactive oxygen species (ROS), predisposing the liver to oxidative damage [10,11,12]. Prolonged use of TMX has been linked with hepatotoxicity, impaired mitochondrial function, and impaired β-oxidation of fatty acids resulting in hepatic steatosis [13,14,15]. In addition to these, TMX is known to also stimulate mitochondrial nitric oxide synthase that increases the intra-mitochondrial peroxynitrite level causing oxidative stress and mitochondrial apoptosis [16].

In recent years, traditional herbal medicine is gaining wider attention both locally and worldwide [17,18]. The interest in herbs and natural products is due to the multiple pharmacological actions they exert on various kinds of diseases [19]. Research efforts are growing in the search for therapeutic agents from medicinal plants. These include phytochemical profiling and isolation of bioactive compounds from herbs as well as the development of multi-herbal formulations through applications of chemical, biochemical, molecular-level investigations and clinical trials [20,21].

*B. coriacea* seed, also known as wonderful Kolanut, has been widely applied in traditional medicine to enhance the memory, “clean the blood”, strengthen the nervous system, treat different kinds of pain, treat hypertension, prevent premature aging, treat headaches, and boost the immune system, etc. [22]. Data from earlier studies carried out in our laboratory indicated that hydroethanolic extract of (defatted) *B. coriacea* seeds (HEBCS) alleviate inflammation and oxidative stress in acute liver disease model [23]. Moreover, it was found to alleviate insulin resistance, hepatic steatosis and inflammation in high fat diet model of non-alcoholic fatty liver disease [24]. Moreover, LC-MS analysis shows that it contains relevant bioactive polyphenols (Umbelliferone, quercetin, acacetin, naringenin, kaempferol, cinnamic acid, vanillin etc.) and alkaloids (pipecolic acid, piperine, Isotussilagine etc.) [24] Therefore, this study is aimed at investigating the protective effects of HEBCS on hepatic triacylglycerol accumulation, oxidative stress, and inflammation associated with tamoxifen-induced hepatotoxicity in the rat model.

## 2. Materials and Methods

### 2.1. Drug and Chemicals

Tamoxifen citrate was a product of West-Coast Works Ltd., Gujarat, India. *n*-hexane, ethanol, 3,3′-Diaminobenzidine (DAB), *p*-nitrophenyl phosphate (*p*-NPP), sodium acetate, sodium carbonate, glutathione were from Merck^®^, Darmstadt, Germany. Guanidine hydrochloride was a product of AK Scientific^®^, Union City, CA, USA. Other chemicals and reagents used were of research grade.

### 2.2. Assay Kits and Antibodies

ELISA Kit for Rat Tumor Necrosis Factor Alpha (TNF-α) was procured from Elabscience^®^ Biotechnology Co. Limited, Houston, TX, USA. Assay kits for alanine aminotransferase (ALT), aspartate aminotransferase (AST), total cholesterol (TC), triglycerides (TG), HDL/LDL-cholesterol are products of Fortress^®^ diagnostics limited, Antrim, UK. Antibodies used for immunohistochemistry assays: Interleukin 6 (IL6), and cyclooxygenase 2 (COX-2), were products of Elabscience^®^ Biotechnology Co. Limited, Houston, TX, USA.

### 2.3. Buchholzia Coriacea Seeds

**Figure 2:**
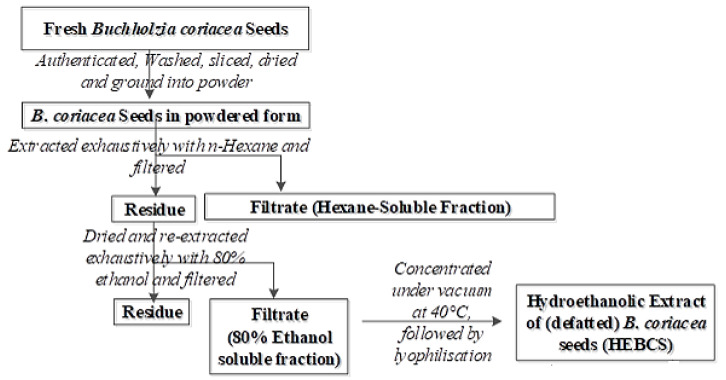
Steps in the preparation of hydroethanolic extract of *Buchholzia coriacea* seeds adapted from Ore et al. [24].

*B. coriacea* seeds were appropriately authenticated as described earlier by Ore et al. [24] Hydroethanolic extract of (defatted) *B. coriacea* seed (HEBCS) was prepared as illustrated in Figure 2.

### 2.4. Experimental Animals

Albino rats (Wistar Strain) used in this research were obtained from the experimental animal breeding house, College of Basic Medical Sciences, University of Ibadan, Oyo State, Nigeria. They were contained in wire-meshed cages and given commercially available rat diet (Ladokun Feeds, Ibadan, Oyo, State Nigeria) with access to water ad libitum. Experimental animal handling agrees with relevant international guidelines on the care and use of laboratory animals in research. This study was approved by the Faculty of Natural Sciences Ethical Review Committee (FNS/ERC/201700016B), Ajayi Crowther University, Oyo, Oyo State, Nigeria.

### 2.5. Experimental Design

**Figure 3:**
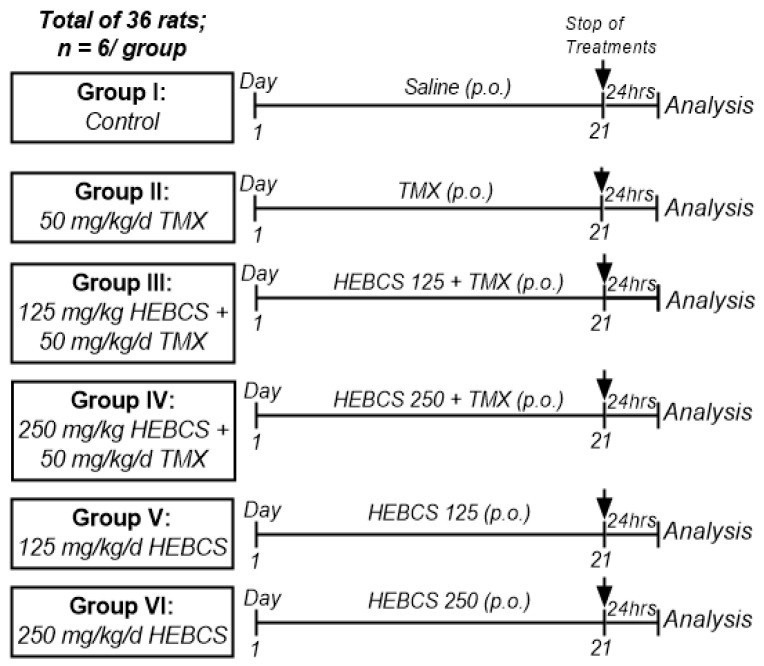
Experimental protocol. HEBCS, hydroethanolic extract of (defatted) B. coriacea seeds; TMX, tamoxifen.

Thirty-six (36) male albino rats (180–260 g; 11–13 weeks old) were assigned into six treatment groups (*n* = 6/group). Rats were acclimatized to laboratory conditions one week before the study commenced. TMX was suspended in physiological saline as previously described [25] and administered at a single dose of 50 mg/kg orally (p.o.) once daily. HEBCS was dissolved in physiological saline at doses of 125 and 250 mg/kg bw. The doses of HEBCS used were selected on the basis of previous studies conducted in our laboratory [23,26]. All treatments were administered as illustrated in Figure 3.

### 2.6. Sample Collection

Following the last administration, rats were fasted overnight and blood samples were collected via the retro-orbital vein in plain sample tubes for preparation of serum. Rats were thereafter euthanized by cervical dislocation and the liver was excised and rinsed in ice-cold phosphate-buffered saline (PBS) (pH 7.4) to remove residual blood. Each liver was blotted until dry and was then weighed. A section from the liver was fixed in 10% neutral-buffered formalin (NBF) for histopathology; an extra section of the liver was cut for the preparation of the frozen section (for oil red O staining) and the remainder was used for the preparation of liver homogenate.

Blood samples were allowed to clot at room temperature and thereafter were subjected to centrifugation at 4000 rpm. for 5 min to obtain serum. Liver sample (0.5 g) was minced and homogenized in PBS (10% *w*/*v*). The homogenate was centrifuged at 10,000× *g* for 10 min at 4 °C. The resulting supernatant was collected and stored frozen until used for biochemical analysis. Protein contents of samples (serum and liver homogenate) was determined using the biuret method [27].

### 2.7. Biochemical Analysis and Immunohistochemistry

Relative liver weight was calculated and serum activities of alanine aminotransferase (ALT) and aspartate aminotransferase (AST) were determined using assay kits (Fortress^®^, Antrim, UK), according to the manufacturer’s protocol. Alkaline phosphatase (ALP) activity was determined by the method of Wright et al. [28]

Serum total cholesterol, triglycerides, HDL- and LDL- cholesterol were determined using assay kits (Fortress Diagnostics Ltd., Atrim, UK) following the manufacturer’s procedure. Hepatic levels of total cholesterol and triglycerides were also determined using assay kits (Fortress Diagnostics Ltd., Atrim, UK).

The hepatic concentration of TNF-α was determined by ELISA kit (Elabscience Biotechnology) following the manufacturer’s procedure. Hepatic expression of IL-6 and COX-2 were evaluated by immunohistochemistry technique as previously described [29].

Nitric oxide (NO) level was determined by the procedure of Green et al. [30] The level of lipid peroxidation (LPO) was evaluated by measuring the concentration of malondialdehyde (MDA) in the serum and liver following the method of Varshney and Kale [31]. Hepatic level of protein carbonyls was determined by the method of Reznick and Packer [32].

Hepatic level of reduced glutathione (GSH) was evaluated based on the method described by Jollow et al. [33] Activity of superoxide dismutase (SOD) in liver was determined according to Sun and Zigman [34]. The method described by Hadwan and Abed [35] was followed to determine the activity of catalase (CAT) in the liver samples. Hepatic glutathione S-transferase (GST) activity was determined by the method of Habig et al. [36], while the method of Rotruck et al. [37] was followed to evaluate hepatic glutathione peroxidase (GSH-Px) activity.

### 2.8. Histopathology

Liver sections previously fixed in NBF were processed for Hematoxylin and Eosin staining as described previously [38]. Oil red O staining was carried out on frozen sections according to the method described by Mehlem et al. [39] Frozen fresh samples were cut in cryostat and air-dried on slides for 30 min and fixed in 10% neutral buffered formalin for 10 min. The slide was rapidly dipped in 60% isopropanol followed by staining in Oil Red O solution for 15 min. The slide was quickly dipped in 60% isopropanol once and then dipped in deionized water. A coverslip was placed with aqueous mounting gel and the image was captured with a light microscope.

### 2.9. Statistical Analysis

The results are expressed as the mean ± SD (*n* = 6). Data were subjected to one-way analysis of variance (ANOVA) and complemented with Tukey’s test (at *p* < 0.05). Statistical analysis and graphical constructions were performed on Graphpad^®^ Prism 6.0.1 (Graphpad Software, La Jolla, CA, USA).

## 3. Results

### 3.1. Variations in Body Weight of Rats

**Figure 4:**
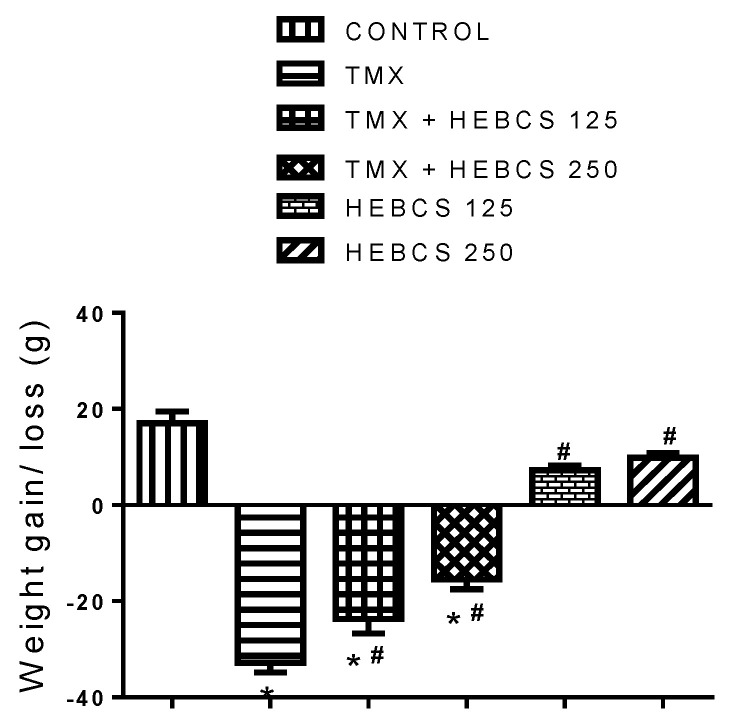
Weight gain/loss in rats following administration of TMX and HEBCS for three weeks. Data are presented as mean ± SD (*n* = 6); * *p* < 0.05 Control versus other groups; # *p* < 0.05 TMX versus HEBCS groups. TMX: Tamoxifen; HEBCS 125 and 250: Hydroethanolic Extract of *Buchholzia coriacea* Seeds, 125 and 250 mg/kg body weight.

Figure 4 shows the changes in the body weight of rats following the administration of TMX and various doses of HEBCS for three weeks. Compared to control, TMX administration caused a significant loss of weight in rats by 174%. A similar decrease in weight was also observed in the animals co-administered with HEBCS, although the decrease in weight in these groups were minimal compared with those in the TMX group. Compared with the TMX group, there was a decrease in weight loss in the groups co-treated with HEBCS 125 and HEBCS 250 by 20% and 36% respectively.

### 3.2. HEBCS Alleviates TMX-Induced Alteration in Liver Function Indices

**Figure 5:**
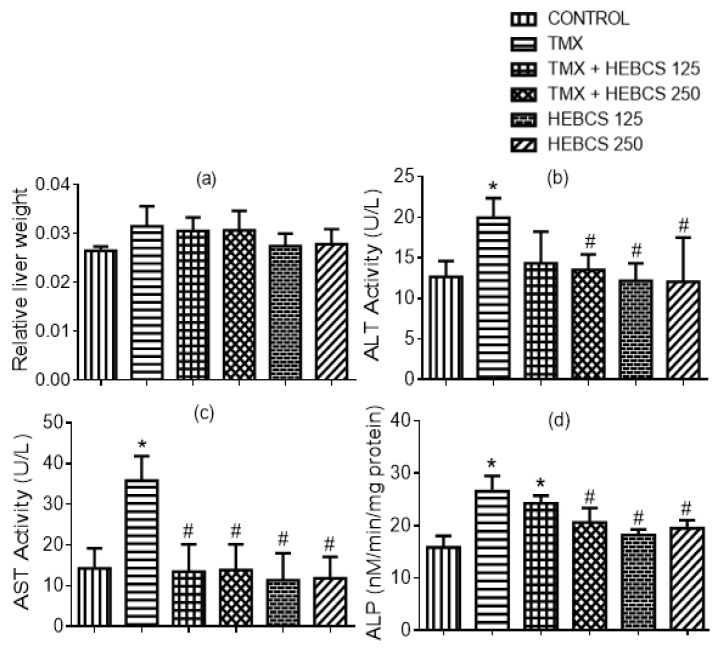
Effect of administration of TMX and HEBCS on (**a**) relative liver weight; (**b**) serum alanine aminotransferase (ALT) activity; (**c**) serum aspartate aminotransferase (AST) activity; (**d**) serum alkaline phosphatase (ALP) activity. Data are presented as mean ± SD (*n =* 6). * p < 0.05 Control versus other groups; # *p* < 0.05 TMX versus HEBCS groups. TMX: Tamoxifen; HEBCS 125 and 250: Hydroethanolic Extract of *Buchholzia coriacea* Seeds, 125 and 250 mg/kg body weight.

TMX administration caused alterations in liver function indices-relative liver weight, and serum activities of ALT, AST and ALP in rats. When compared with the control group, the TMX group demonstrated a slight increase in relative liver weight by 15% (Figure 5a) although this was not significant (*p* < 0.05). There was a significant increase (*p* < 0.05) in the activities of ALT, AST and ALP in the serum of rats in the TMX group by 57%, 60% and 68% respectively when compared with the control (Figure 5b–d). However, administering HEBCS at 125 and 250 mg/kg alongside TMX alleviated the TMX–induced increase in serum ALT, AST and ALP activities by 21% and 28%, 62% and 61% and 9% and 23% respectively. When compared with TMX, a statistically significant decrease (in serum activities of ALT, AST and ALP) was observed in the TMX + HEBCS 250 group. The protection exerted by the lower dose of HEBCS (HEBCS 125) against TMX-induced increases in the serum activities of these enzymes was only significant in the case of AST.

### 3.3. HEBCS Alleviates TMX-Induced Alteration in Lipid Profile in Rats

**Figure 6:**
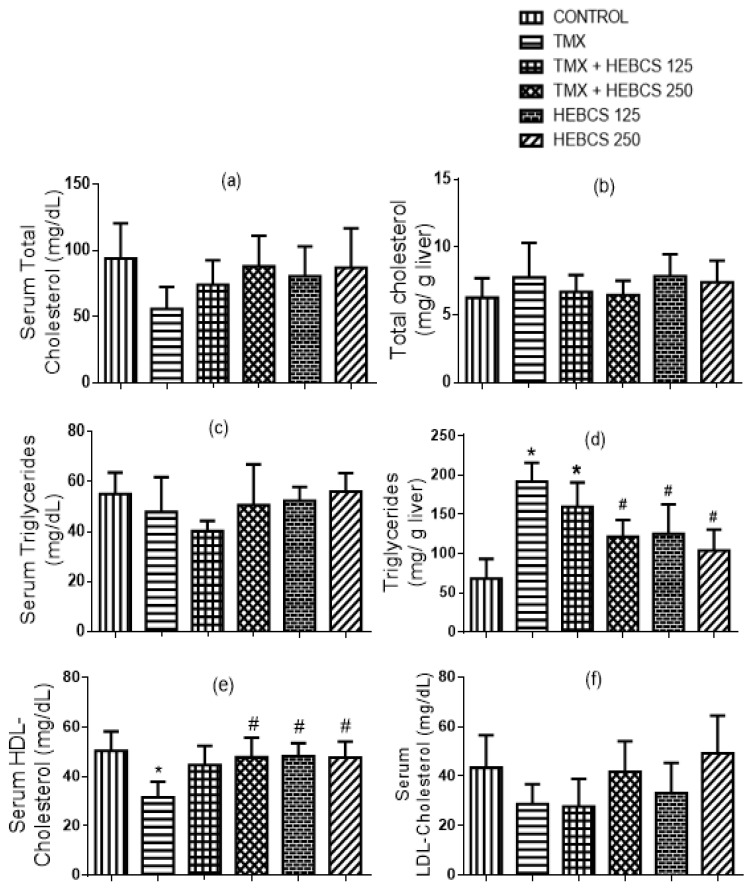
Ameliorative effect of HEBCS against TMX–induced alteration in serum and hepatic lipids concentrations (**a**) serum total cholesterol; (**b**) hepatic total cholesterol; (**c**) serum triglycerides; (**d**) hepatic triglycerides; (**e**) serum high density lipoprotein-cholesterol (HDL-cholesterol); (**f**) serum low density lipoprotein-cholesterol (LDL-cholesterol). Data are presented as mean ± SD (*n* = 6). * *p* < 0.05 Control versus other groups; # *p* < 0.05 TMX versus HEBCS groups. TMX: Tamoxifen; HEBCS 125 and 250: Hydroethanolic Extract of *Buchholzia coriacea* Seeds, 125 and 250 mg/kg body weight.

TMX treatment caused no significant change in the serum and hepatic cholesterol levels in rat (Figure 6a,b). There was also no significant change in serum triglyceride level (Figure 6c). In contrast to the effect of TMX on serum triglycerides, TMX treatment significantly increased the hepatic triglycerides level by 166% when compared with the control. However, co-administration of HEBCS with TMX at 250 mg/kg significantly decreased the elevated hepatic triglycerides level by 26% and reversed the TMX-induced increase in the hepatic triglycerides level (Figure 6d). When compared with control, a statistically significant decrease in serum HDL-cholesterol level (by 37%) was observed following TMX administration in rats (Figure 6e). On the other hand, serum LDL-cholesterol level did not change significantly following TMX administration (Figure 6f). However, co-treatments with HEBCS ameliorated the TMX-induced decrease in HDL-cholesterol where the protective effect exerted by only HEBCS 250 was statistically significant (*p* < 0.05).

### 3.4. HEBCS Alleviates TMX-Induced Increase in Hepatic Levels of Pro-Inflammatory Markers

**Figure 7:**
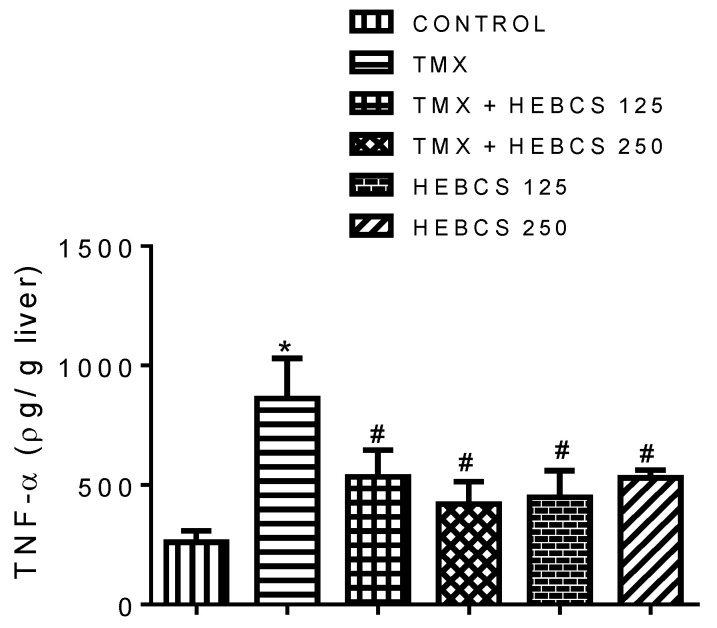
Ameliorative effect of HEBCS against TMX-induced increase in hepatic TNF-α concentration in rats. Data are presented as mean ± SD (*n* = 6). * *p* < 0.05 Control versus other groups; # *p* < 0.05 TMX versus HEBCS groups. TNF-α: tumour necrosis factor alpha; TMX: Tamoxifen; HEBCS 125 and 250: Hydroethanolic Extract of *Buchholzia coriacea* Seeds, 125 and 250 mg/kg body weight.

**Figure 8:**
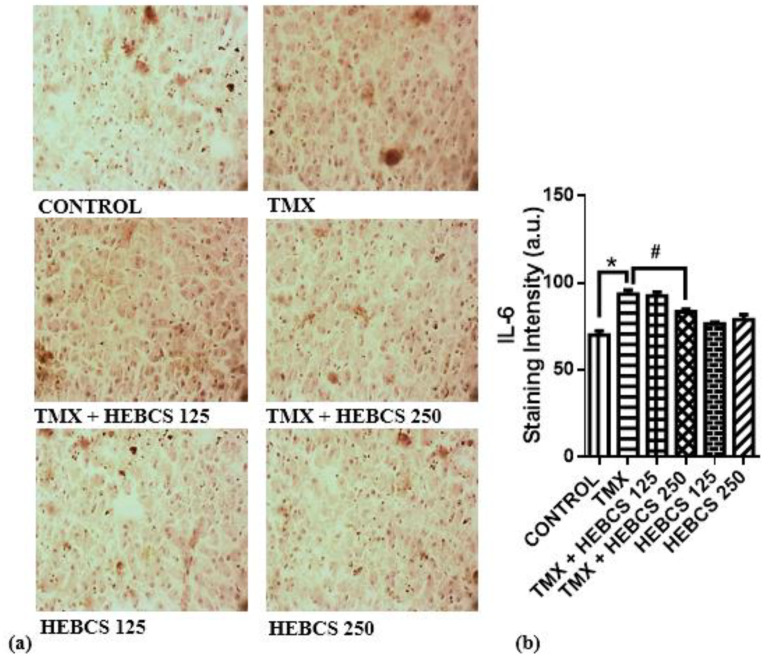
Immunohistochemistry slides (×200) (**a**) showing the hepatic expression of IL-6 in rats. Bar chart in (**b**) shows the staining intensity of each slide. Staining intensity of positive areas was measured using ImageJ. Data are presented as mean ± SD (*n* = 6). * *p* < 0.05 Control versus other groups; # *p* < 0.05 TMX versus HEBCS groups. IL-6: interleukin-6; TMX: Tamoxifen; HEBCS 125 and 250: Hydroethanolic Extract of *Buchholzia coriacea* Seeds, 125 and 250 mg/kg body weight.

**Figure 9:**
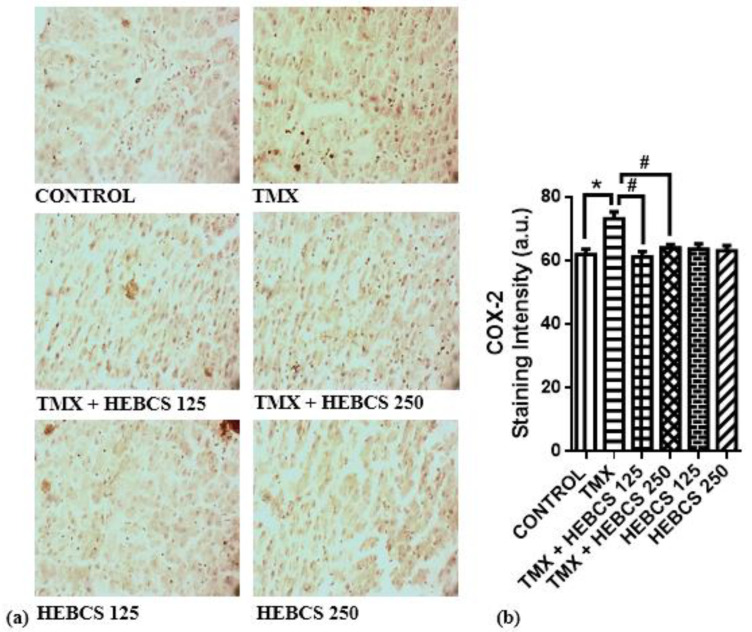
Immunohistochemistry slides (×200) (**a**) showing the hepatic expression of COX-2 in rats. Bar chart in (**b**) shows the staining intensity of each slides. Staining intensity of positive areas was measured using ImageJ. Data are presented as mean ± SD (*n* = 6). * *p* < 0.05 Control versus other groups; # *p* < 0.05 TMX versus HEBCS groups. COX-2: cyclooxygenase 2; TMX: Tamoxifen; HEBCS 125 and 250: Hydroethanolic Extract of *Buchholzia coriacea* Seeds, 125 and 250 mg/kg body weight.

A significant increase in the level of pro-inflammatory markers—TNF-α, COX-2 and IL-6—was observed following the administration of TMX to rats. Hepatic concentration of TNF-α increased significantly (*p* < 0.05) in the TMX group by 220% when compared with the control (Figure 7). However, administration of HEBCS at 125 and 250 mg/kg alleviated this TMX-induced increase in hepatic concentration of TNF-α by 38% and 75% respectively. (Figure 8a) and (Figure 9a) shows the hepatic expression of IL-6 and COX-2 following administration of TMX and HEBCS in rats; bar (Figure 8b) and (Figure 9b) show the corresponding staining intensities. As shown in (Figure 8b) and (Figure 9b), there was a significant increase (*p* < 0.05) in hepatic expression of IL-6 and COX-2 in the TMX-treated group when compared with the control. In Figure 8, both HEBCS 125 and HEBCS 250 significantly protected against TMX-induced increases in hepatic expression of IL-6. Similarly, in Figure 9, HEBCS 250 significantly reversed the TMX-induced increase in hepatic expression of COX-2 in rats, whereas the protective effect exerted by HEBCS 125 was not significant compared with the TMX group.

### 3.5. HEBCS Alleviates a TMX-Induced Increase in Levels of Markers of Hepatic Oxidative Stress

**Figure 10:**
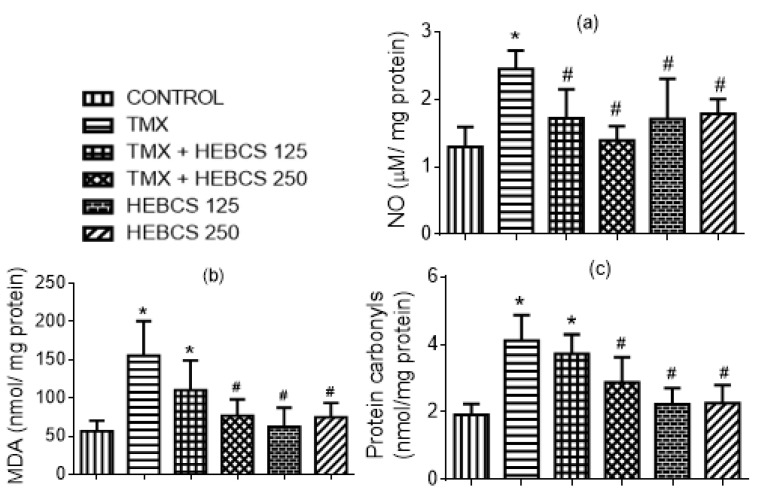
Ameliorative effect of HEBCS against TMX induced an increase in hepatic (**a**) NO level; (**b**) MDA level and (**c**) protein carbonyls level in rats. Data are presented as mean ± SD (*n* = 6). * *p* < 0.05 Control versus other groups; # *p* < 0.05 TMX versus HEBCS groups. MDA: malondialdehyde; NO: nitric oxide; TMX: Tamoxifen; HEBCS 125 and 250: Hydroethanolic Extract of Buchholzia coriacea Seeds, 125 and 250 mg/kg body weight.

Figure 10 shows the protective effects of HEBCS against TMX-induced increase in hepatic levels of biomarkers of oxidative stress (NO, MDA and protein carbonyls) in rats. TMX caused a significant increase (*p* < 0.05) in hepatic nitric oxide level by 89% when compared with the control (Figure 10a). Similarly, hepatic MDA and protein carbonyls increased significantly in the TMX-treated animals by 175% and 114% respectively. However, co-administration with HEBCS significantly protected against TMX-induced increases in hepatic NO levels. Co-administration of HEBCS alongside TMX also reversed the TMX-induced elevation in hepatic protein carbonyls and MDA in rats (Figure 10b,c). As shown in Figure 10b,c, HEBCS 250 exerts a statistically significant protection against TMX-induced increases in hepatic MDA and protein carbonyls in rats.

### 3.6. HEBCS Alleviates TMX-Induced Depletion of Hepatic Antioxidants

**Figure 11:**
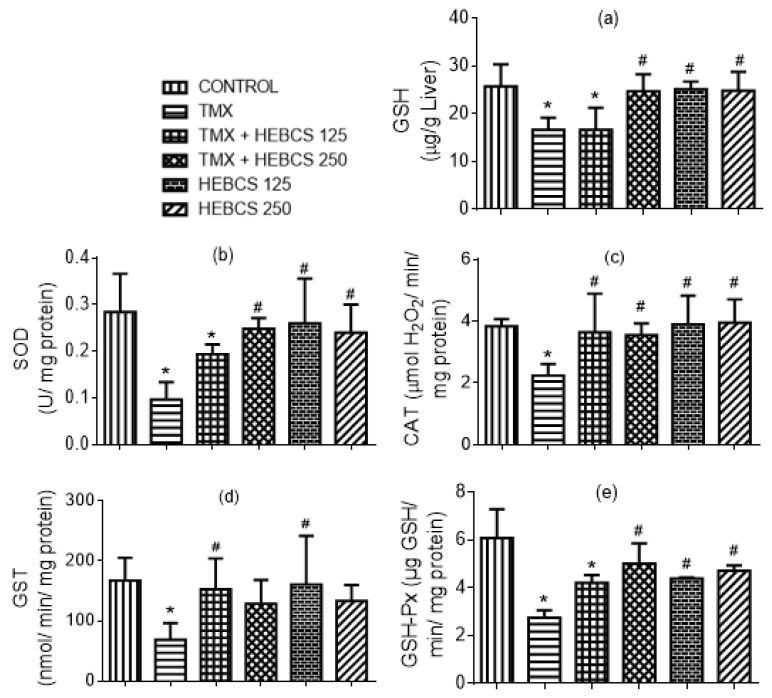
Ameliorative effect of HEBCS against TMX-induced decrease in (**a**) hepatic GSH level; (**b**) hepatic SOD activity; (**c**) hepatic CAT activity; (**d**) hepatic GST activity; and (**e**) hepatic GSH-Px activity in rats. Data are presented as mean ± SD (*n* = 6). * *p* < 0.05 Control versus other groups; # *p* < 0.05 TMX versus HEBCS groups. GSH: reduced glutathione; SOD: superoxide dismutase; CAT: catalase; GST: glutathione S-transferase; GSH-Px: glutathione peroxidase; TMX: Tamoxifen; HEBCS 125 and 250: Hydroethanolic Extract of *Buchholzia coriacea* Seeds, 125 and 250 mg/kg body weight.

To assess the hepatoprotective effects of HEBCS on TMX-induced depletion of hepatic antioxidants, the levels of the non enzymic antioxidants, GSH level as well as hepatic activities of enzymic antioxidants, superoxide dismutase (SOD), catalase (CAT), glutathione S-transferase (GST) and glutathione peroxidase (GSH-Px), were evaluated. Hepatic concentration of GSH decreased following TMX administration in rats by 35% (Figure 11a). In a similar manner, hepatic activities of SOD, CAT, GST and GSH-Px decreased in response to TMX treatments in rats by 67%, 41%, 59% and 53% respectively (Figure 11b–e). Co-administration of HEBCS alongside TMX protected against the TMX-induced depletion of hepatic antioxidants. In Figure 11a, only HEBCS 250 was able to significantly reverse the TMX-induced decrease in hepatic GSH level. Co-administration of HEBCS alongside TMX also protected against the TMX-induced decrease in hepatic activities of SOD, CAT, GST and GSH-Px in rats. In all the enzymic antioxidants determined, HEBCS 250 effectively reversed the effects of TMX except in the case of GST, where only HEBCS 125 showed a significant protection against TMX.

### 3.7. HEBCS Alleviates TMX-Induced Alteration in Hepatic Histological Structure

**Figure 12:**
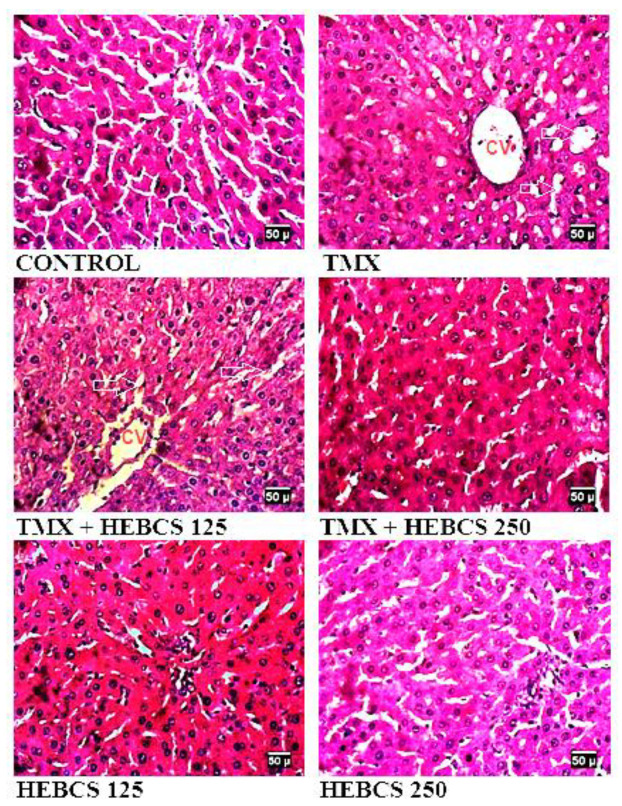
Representative images (×400) of hematoxylin and eosin stained formalin-fixed paraffin-embedded liver sections showing the effects of HEBCS and TMX on hepatic structures. CV: central vein; TMX: Tamoxifen; HEBCS 125 and 250: Hydroethanolic Extract of *Buchholzia coriacea* Seeds, 125 and 250 mg/kg body weight.

Slides in Figure 12 are representative images (×400) of hematoxylin and eosin stained formalin-fixed paraffin-embedded liver sections, showing the protective effect of HEBCS on TMX-induced hepatic degeneration in rats. Control slide shows normal histological appearance while that labelled with TMX shows Grade 3 hepatocellular ballooning (swelling) and vacuolar degeneration (arrows). TMX treated with HEBCS 125 displays Grade 1 hepatocellular ballooning and vacuolar degeneration (arrows). However, images labelled TMX + HEBCS 250, HEBCS 125 and HEBCS 250 shows the relatively normal histological appearance of the liver.

**Figure 13:**
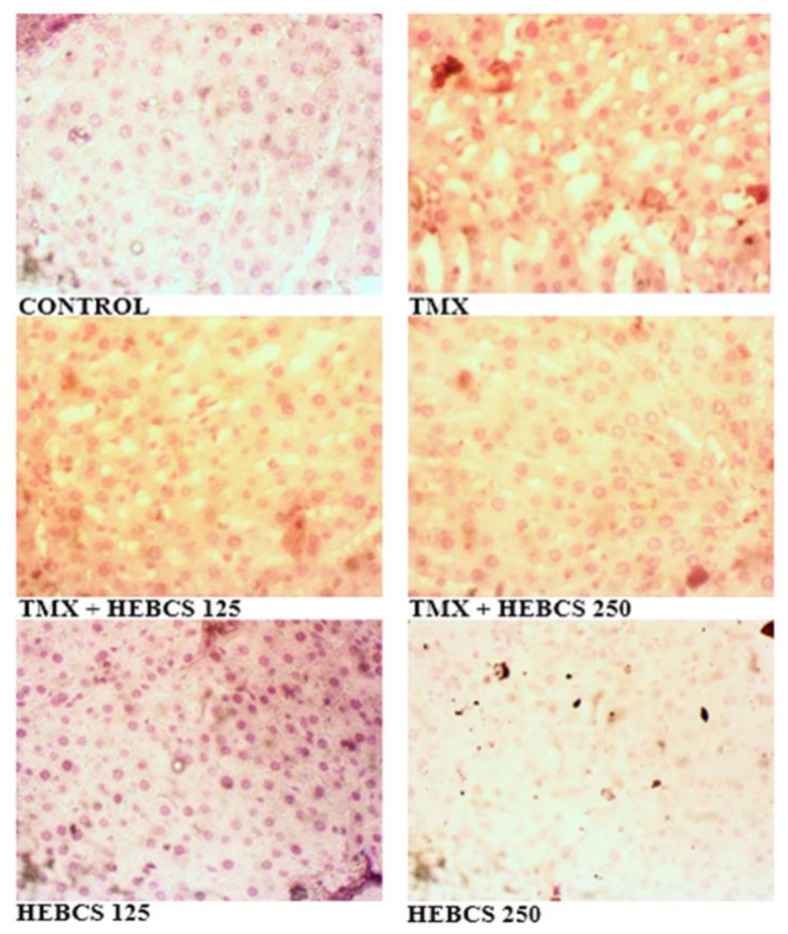
Representative images (×400) of Oil red O stained liver sections. TMX: Tamoxifen; HEBCS 125, 250: Hydroethanolic Extract of *Buchholzia coriacea* Seeds, 125 and 250 mg/kg body weight.

Slides in Figure 13 are the representative images of Oil red O (ORO) stained liver sections showing the protective effects of HEBCS on TMX–induced accumulation of triacylglycerol in rats. The control slide is negative for ORO, while TMX and TMX + HEBCS 125 shows grade 3 positivity for ORO. However, the group treated with HEBCS 250 shows grade 2 positivity, indicating some degree of protection by HEBCS 250.

**Figure 14:**
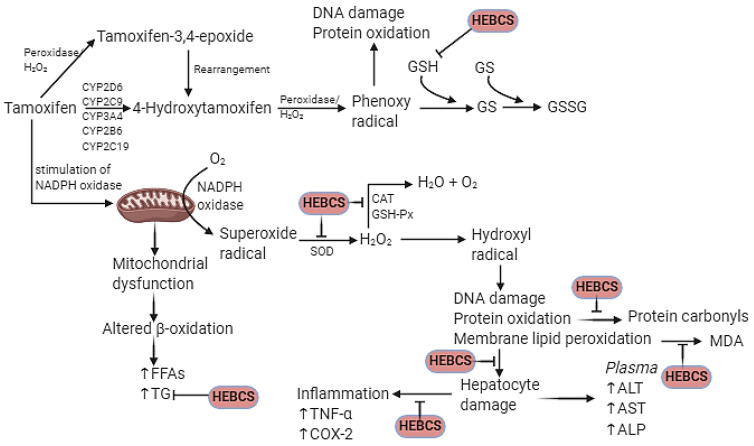
Proposed mechanism by which HEBCS alleviate Tamoxifen induced hepatotoxicity in rat (created at www.BioRender.com, accessed on 4 August 2021). CYP, cytochrome P450; TNF-α, tumour necrosis factor alpha; FFA, free fatty acid; HEBCS, hydroethanolic extract of Buchholzia coriacea seeds; ALT, alanine aminotransferase; AST, aspartate aminotransferase; ALP, alkaline phosphatase.

Figure 14 shows the mechanistic scheme proposed for the protective effects of HEBCS on TMX-induced hepatotoxicity in rat. The protective mechanism of action displayed by HEBCS include antioxidant, anti-inflammatory and anti-hyperlipidemic.

## 4. Discussion

Drugs are gradually becoming a significant cause of liver injury in the general population and drug-induced hepatic injury (DILI) is currently one of the most important reasons for drug withdrawals at the clinical level. Tamoxifen (TMX) is an antiestrogen widely used in the treatment and prevention of all stages of estrogen-dependent breast cancer. However, TMX is associated with adverse effects, mainly oxidative stress and hepatotoxicity often presented as hepatitis, hepatic steatosis, hepatocyte ballooning, hepatic necrosis, hepatocarcinoma, etc. [11,12,13,14,15] *Buccholzia coriacea* seeds (BCS) have been widely applied in traditional medicinal practice to treat numerous diseases. BCS has also been evaluated at an experimental level with evidence-based pharmacological actions including immunomodulatory, anti-inflammatory, antioxidant, and anti-hyperlipidemia activity among others [23,40,41,42]. The present study explores the antihyperlipidemic, anti-inflammatory and antioxidant properties of HEBCS against hepatocellular damage, hepatic lipid accumulation, inflammation and oxidative stress associated with TMX.

Data from this study show that TMX treatment (50 mg/kg/d) for twenty-one days caused a significant decrease in body weight. The decrease in body weight observed may be related to the high dosage of TMX used in this study, which differs from the dosage used for the long-term TMX treatment of breast cancer patients. However, a recent study reported a decrease in weight as a result of TMX treatment in mice, which was attributed to a reduction in fat mass [43]. This decrease in fat mass may be attributed to a number of cellular processes including apoptosis and autophagy [44,45] (processes that reduce adipocyte number) and significant ROS generation by TMX [12,43]. Co-administration of HEBCS alongside TMX in this study slightly alleviate the observed TMX-induced decrease in body weight in rats.

Our data demonstrated that TMX administration resulted in significant elevation of serum activities of ALT, AST, and ALP in rats. These results are consistent with those reported by Qasim and Baraj [25] where 50 mg/kg TMX caused hepatotoxicity in albino rats. TMX has been reported to induce oxidative liver damage and produce liver injury with elevation in plasma or serum levels of liver function biomarkers like ALT, AST and ALP [46,47]. The pattern of elevation of these markers has been shown to be vital to the diagnosis of the type of liver injury involved [48]. The aminotransferases (ALT and AST) are biomarkers of hepatocellular injury. They catalyze the transfer of amino groups from alanine or aspartate to ketoglutarate to produce pyruvate and oxaloacetate respectively. AST is found in the liver and other organs like kidneys, brain, pancreas, lungs, and cardiac muscle, while ALT is found in high concentrations in the liver. Hepatocellular damage often results in the release of these enzymes into the circulation [48]. ALP is a zinc metalloenzyme which is present in high concentrations in the bile canaliculus as well as in other tissues. Increase in serum activity of ALP is associated with hepatobiliary and cholestatic injury [48,49]. The alterations in serum activities of the liver function biomarkers induced by TMX were significantly improved with co-administration of HEBCS to TMX-intoxicated rats. A similar hepatoprotective effect of BCS has been reported by Okolie et al. [50] where butanol fraction of BCS extract protected against the streptozotocin-induced increase in serum AST, ALT, and ALP activities in Wistar rats.

TMX treatment also caused a significant increase in hepatic triglycerides and a decrease in serum HDL-cholesterol level, but no significant change in serum and hepatic total cholesterol, serum triglycerides and LDL-cholesterol. This observation is consistent with those reported earlier by Behrouj et al. [51], Cole et al. [52] and Gudbrandsen et al. [53] Tamoxifen-induced hepatic TG accumulation (fatty liver) has been observed in breast cancer patients undergoing TMX chemotherapy [54]. TMX-induced hepatic steatosis has been linked to mitochondrial dysfunction and impaired β-oxidation of fatty acids [55]. Data from this study show that HEBCS protected against TMX-induced elevation in hepatic TG level and alterations in serum lipid profile. This protection may be attributed to the anti-dyslipidemic effects of BCS as reported earlier [42].

Cytokines like TNF-α and interleukin 6, as well as an inducible enzyme like COX-2, are established pro-inflammatory biomarkers. Their concentrations or expressions are often used to assess inflammatory events in tissues. Data from this study show an elevated hepatic level of TNF-α in rats treated with TMX. Earlier report by El-Beshbishy et al. [56] revealed an elevated serum level of TNF-α in response to 45 mg/kg/day TMX treatment in rats. Furthermore, a similar study by Suddek [57] also showed a significant increase in hepatic TNF-α level in response to 45 mg/kg/day TMX treatment. We also observed a significant increase in hepatic expression of IL-6 and COX-2 following TMX treatment in rats. While there are limited or no information on the relationship between TMX treatment and hepatic IL-6 expression, earlier reports have shown that COX-2 may play a vital role as a predictor of adverse effects of TMX in breast cancer patients [58]. Our data show that co-administration of HEBCS alongside TMX significantly alleviate the observed TMX-induced elevation of hepatic inflammatory markers. These results are consistent with an earlier report on the anti-inflammatory activity exhibited by HEBCS against LPS-induced inflammation in rats [23].

TMX treatment in this study leads to a significant increase in hepatic oxidative stress biomarkers. This is evident by the observed increase in hepatic NO level, MDA (a marker of oxidative damage to lipids) and hepatic protein carbonyls (products of protein oxidation). TMX has been shown to be associated production of ROS such as superoxide radicals and NO [12,16]. NO is produced via an increase in expression of nitric oxide synthase II (NOS2) [59]. Overproduction of NO and other ROS generated during the oxidative metabolism of TMX contributes to an increase in lipid peroxidation and protein oxidation as indicated by the elevated hepatic level of MDA and protein carbonyls in this study. Current observations of TMX-induced increase in hepatic NO, MDA and protein carbonyls is consistent with previous reports by Albukhari et al. [46] and Tabassum et al. [60] Our data show that co-administration of HEBCS alongside TMX significantly alleviates TMX-induced oxidative stress as indicated by a decrease in hepatic NO, MDA and protein carbonyl levels in rats. In contrast to the elevation in hepatic NO, MDA and protein carbonyls in the TMX-induced group, concentrations of these oxidative stress products in the HEBCS-treated groups were found to be close to normal, underscoring antioxidant protection offered by HEBCS. These data suggest the ability of HEBCS to significantly combat oxidative stress. Suppression of oxidative stress by HEBCS in the present study is consistent with an earlier report [23].

Additionally, TMX administration in this study caused a significant depletion of the hepatic antioxidant defense system in rats. Hepatic GSH level and activities of SOD, CAT, GST, and GSH-Px decreased significantly in TMX-treated rats. GSH is a non-enzymic antioxidant, often the first line defense against oxidants in vivo. SOD plays a role in the dismutation of superoxide radicals to H_2_O_2_, another oxidant and a substrate for CAT and GSH-Px. GST requires the presence of GSH for activity and it participates in the detoxification of drugs and toxicant. A decrease in the activities of SOD, CAT, and GSH-Px may lead to accumulation of superoxide radicals and H_2_O_2_ in hepatocytes, which may be responsible for the observed increase in hepatic oxidants and oxidative products in the TMX group. A high level of oxidants can lead to membrane lipid peroxidation, thereby damaging the hepatocytes. Our data show that administration of HEBCS, along with TMX, significantly alleviates oxidative stress induced by TMX by improving hepatic antioxidant status in rats. Improvement in the hepatic antioxidant system by HEBCS against TMX in the present study agrees with an earlier report on the effect HEBCS against LPS-induced oxidative stress [23].

Our data also indicated that TMX induced histopathological changes in liver tissues. TMX treatment caused hepatocellular ballooning and vacuolar degeneration as indicated in the H&E stained sections; ORO staining also indicated the presence of fat deposits in sections from the TMX group. TMX has been reported to induce significant hepatic steatosis and hepatocellular ballooning [46]. HEBCS administration, alongside TMX, significantly improved the histological degeneration caused by TMX.

## 5. Conclusions

Findings from the present study indicate that HEBCS protects against TMX-induced hepatotoxicity via anti-dyslipidemic, antioxidant and anti-inflammatory actions. HEBCS may be of therapeutic potential in alleviating the hepatotoxic effects of chemotherapeutic agents. It may be useful as a component of combination therapy in cancer patients receiving TMX treatment. It may also protect against other forms of liver injury involving steatosis, inflammation, free radicals and oxidative damage.

## Data Availability

Not applicable.
